# M2 macrophages activate the IL-10/JAK2/STAT3 pathway to induce pathological microangiogenesis in the nucleus pulposus exacerbating intervertebral disc degeneration

**DOI:** 10.1186/s13018-025-05962-2

**Published:** 2025-05-28

**Authors:** Si-ping Zhang, Min Tong, Jun Mo, Zhen-Yu Dong, Yi-Fei Huang

**Affiliations:** 1 The Fourth Clinical Medical College of Xinjiang Medical University, Urumqi, Xinjiang 830000 P.R. China; 2 Department of Spinal Surgery, Traditional Chinese Medicine Hospital, Xinjiang Medical University, Urumqi, Xinjiang 830000 P.R. China; 3 Xinjiang Uygur Autonomous Region Academy of Traditional Chinese Medicine, Urumqi, Xinjiang 830000 P.R. China

**Keywords:** Intervertebral disc degeneration, Macrophage, Microangiogenesis, Nucleus pulposus

## Abstract

**Background:**

Macrophage infiltration accompanied by pathological microangiogenesis in the nucleus pulposus (NP) plays a critical role in the progression of intervertebral disc degeneration (IDD). However, the involvement of M2 macrophages in mediating NP pathological angiogenesis and their underlying mechanisms remain unclear.

**Methods:**

Firstly, the expression of M2 macrophage (CD206) and microangiogenic (CD34) markers in human degenerated NP was observed by immunohistochemical staining, subsequently, a co-culture system of M2 macrophages and NP cells was established. IL-10 expression was silenced using siRNA to assess the pro-angiogenic effects of M2 macrophages in IDD via IL-10 and its downstream janus kinase (JAK) 2/ signal transducer and activator of transcription (STAT) 3 pathway. AG490, a specific JAK2/STAT3 inhibitor, was applied to determine whether IL-10 exerts its effects through this pathway and to evaluate its impact on angiogenesis and extracellular matrix (ECM) metabolism in NP pathology.

**Results:**

CD206 and CD34 were co-expressed in degenerated NP tissue. Degenerated NP cells secreted CCL17, CCL18, and CD206, exhibiting M2-like characteristics. Co-culture of M2 macrophages with degenerated NP cells led to IL-10 secretion to promote CD34 expression, and downregulated anabolic genes (type II collagen (COL2), aggrecan), and upregulated catabolic genes (matrix metalloproteinase (MMP)-3, MMP-7). JAK2 and STAT3 expression was significantly increased following co-culture. Activation of the JAK2/STAT3 pathway enhanced vascular endothelial growth factor (VEGF), vascular endothelial growth factor receptor (VEGFR), and CD34 expression and induced further downregulation of COL2 and aggrecan and upregulation of MMP-3 and MMP-7.

**Conclusion:**

M2 macrophage infiltration and pathological neovascularization are prominent in degenerated NP tissue. IL-10 secreted by M2 macrophages activates the JAK2/STAT3 pathway to promote pathological microangiogenesis by up-regulate the expression of VEGF/VEGFR. This process disrupts ECM and accelerates the progression of IDD.

**Clinical trial number:**

Not applicable.

Low back pain has become a significant global public health concern due to its high prevalence and associated disability, which collectively impose a substantial economic burden on society [1, 2]. Intervertebral disc degeneration (IDD) is a major cause of low back pain [3]. Current clinical interventions for symptomatic IDD remain limited and do not effectively reverse disease progression. The intervertebral disc (IVD), the largest avascular structure in the human body, is particularly vulnerable to pathological changes. Aberrant microangiogenesis within the nucleus pulposus (NP) leads to disc vascularisation (IDV), a process that compromises IVD structural integrity and function, thereby accelerating IDD [4]. Macrophages are the only immune cells capable of infiltrating the NP through the annulus fibrosus, and they play a pivotal role in IDD pathophysiology [5]. While the pro-inflammatory effects of M1 macrophages in promoting IDD progression are well established, the functional role of M2 macrophages remains controversial [6–8]. Relevant studies have shown that M2 macrophages promote microangiogenesis by secreting interleukin-10 (IL-10) during tissue repair, highlighting their potential pro-angiogenic activity [9, 10]. Although IL-10 is commonly recognized as a key anti-inflammatory cytokine, it also activates downstream signaling via Janus kinases (JAKs) - signal transducers and activators of transcription (STATs) [11]. Notably, the JAK2/STAT3 signaling cascade has been implicated in angiogenesis induced by IL-10 [12]. However, whether IL-10 secreted by M2 macrophages within degenerated NP tissues contributes to IDD progression by promoting pathological microangiogenesis through JAK2/STAT3 activation remains unclear. The objective of the present study was to investigate the effect of IL-10 upregulation in M2 macrophages on microvascular proliferation and associated signaling during IDD. In addition, the study aimed to determine whether the resulting angiogenesis disrupts extracellular matrix (ECM) homeostasis, thereby accelerating IDD.

## Materials and methods

### Collection of human IVD

This study was granted by the Ethics Committee of the Affiliated Hospital of Traditional Chinese Medicine of Xinjiang Medical University prior to commencement (Approval No. 2022XE-QZRYS0061). Written informed consent was obtained from all participants. IVD specimens were collected from 15 patients undergoing lumbar discectomy, with all procedures performed by the same surgical team. Degeneration grading was assessed using the modified Pfirrmann grading system [13], identifying one case of grade 2, five cases of grade 3, two cases of grade 4, one case of grade 5, three cases of grade 6, and three cases of grade 7. The cohort included nine males and six females, with a mean age of 38.27 ± 8.10 years. Degenerative changes in the NP were evaluated by immunohistochemical (IHC) staining. The baseline demographic and clinical characteristics of the patients are presented in Table 1.

**Table 1:** The baseline information of the included patients

Indicators	Patients
Sex (Male/Female)	9/6
Age (years)	38.27 ± 8.10
BMI	25.79 ± 3.74
IVD segment	
L4/5	8
L5/S1	7
Modified Pfirrmann grading	
grade 2	1
grade 3	5
grade 4	2
grade 5	1
grade 6	3
grade 7	3

### Sampling, fixation, embedding and sectioning of human NP

IVD specimens were rinsed with phosphate-buffered saline (PBS) to remove residual blood and impurities. Cartilaginous endplates and annulus fibrosus were carefully excised, and the remaining NP tissue was isolated and rinsed again with PBS. Specimens were fixed in 4% paraformaldehyde for 24 h. After fixation, the NP tissues were trimmed, transferred to embedding cassettes, thoroughly washed with distilled water, and dehydrated through a graded ethanol series (70%, 85%, 95%, and 100%). Dehydrated samples were cleared with xylene and then infiltrated with molten paraffin. Paraffin embedding was performed, and the blocks were cooled on ice for 5 h. Once solidified, the blocks were sectioned into 5 μm-thick slices using a microtome and dried in a 60 °C incubator prior to staining.

### IHC staining

Paraffin-embedded tissue sections were first deparaffinized in xylene and rehydrated through a descending ethanol series (100%, 95%, 85%, and 70%). After rinsing with PBS, sections were transferred to antigen retrieval buffer and subjected to heat-induced epitope retrieval by microwave treatment for 10 min, followed by natural cooling and PBS washing. Endogenous peroxidase activity was blocked by incubation with 3% hydrogen peroxide in methanol for 10 min at room temperature, followed by thorough PBS washing. To reduce nonspecific binding, each section was incubated with 50 µL of 1% bovine serum albumin (BSA) at room temperature for 20 min. Primary antibodies were applied by dropwise addition of 50 µL of either anti-CD206 (18704-1-AP, Proteintech) or anti-CD34 (14486-1-AP, Proteintech), and sections were incubated overnight at 4 °C. The following day, 50 µL of HRP-conjugated sheep anti-rabbit/mouse secondary polymer reagent was applied and incubated for 20 min in a humidified chamber at room temperature. Sections were washed in PBS and developed using freshly prepared diaminobenzidine (DAB) solution. After color development was terminated, hematoxylin counterstaining was performed for 10 min, followed by thorough rinsing with distilled water. Sections were then dehydrated through a graded ethanol series (70%, 85%, 95%, and 100%), cleared in xylene, and mounted with neutral gum. Stained sections were examined under a light microscope for histological evaluation.

### Culture of human primary NP cells and induction of degeneration

Human primary NP cells were cultured in a humidified incubator maintained at 37 °C with 5% CO₂. Once the cells reached approximately 80% confluence, they were digested, centrifuged, and resuspended at a concentration of 6 × 10⁴ cells/mL. A lipopolysaccharide (LPS) stock solution (10 mg/mL) was diluted 100-fold, and serial dilutions were then prepared to obtain final concentrations of 16 µg/mL, 8 µg/mL, 4 µg/mL, 2 µg/mL, 1 µg/mL, and 0.5 µg/mL. In a 96-well plate, 100 µL of the NP cell suspension was added to each well. After cell adhesion, an equal volume (100 µL) of medium containing the respective LPS concentration was added. Cells were incubated for 24 h to induce degeneration.

### Polarisation of macrophages

THP-1 cells were cultured in RPMI-1640 medium supplemented with 10% fetal bovine serum (FBS) and 1% penicillin-streptomycin in a humidified incubator at 37 °C with 5% CO₂. When cell density reached 1 × 10⁵ cells/mL, phorbol 12-myristate 13-acetate (PMA) was added for 48 h to induce differentiation into adherent macrophage-like cells. Following adherence, interleukin-4 (IL-4) and interleukin-13 (IL-13) were added to the medium to induce M2 macrophage polarisation, and incubation was continued for an additional 48 h.

### Silencing of IL-10 expression by SiRNA transfection

IL-10 gene expression in M2 macrophages was silenced using siRNA transfection. The siRNA sequences targeting IL-10 were as follows: IL-10, 5’-UAAUAAGCUCCAAGAGAAATT-3’ and 5’-UUUCUCUUUUGGAGCUUUAUUUUATT-3; GAPDH, 5’-UUCUUCCGAACGUGUCACGUTT-3’and 5’-ACGUGACACGUUCGGAGAATT-3’.

### Macrophages co-cultured with NP cells

A co-culture system was established utilizing Transwell chambers to investigate the interaction between M2 macrophages and NP cells. NP cells were fully digested and seeded into the lower chamber at a density of 2.5 × 10⁵ cells/mL in 2 mL of medium. M2 macrophages were seeded into the upper chamber at a density of 5 × 10⁵ cells/mL in 1 mL of medium following enzymatic digestion. After 48 h of co-culture, cells were harvested from both compartments for further analysis.

### Cell counting Kit-8 (CCK-8)

Cell viability was assessed using the CCK-8 kit (KGA9306-500, KeyGEN Biotech). A total of 100 µL of culture medium containing 10% CCK-8 reagent was added to each well of the cell culture plate. Plates were incubated for 2 h at 37 °C in a humidified incubator. After incubation, gentle shaking was applied for 10 min to ensure uniform mixing. The absorbance at 450 nm was measured utilizing a microplate reader, and cell survival rates under different LPS concentrations were calculated accordingly.

### Enzyme-linked immunosorbent assay (ELISA)

Interleukin-1β (IL-1β) expression was quantified utilizing an ELISA kit (KGC1103-96, KeyGEN Biotech). Serial dilutions of the standard solution and 100 µL of each sample were added to the corresponding wells of the enzyme-coated plate. An equal volume (100 µL) of enzyme reagent was added to each well, except for the blank controls. The plate was sealed with a sealing film and incubated at 37 °C for 90 min in a thermostatic chamber. Following incubation, 50 µL each of Chromogen Solution A and Chromogen Solution B was added sequentially to each well. The plate was incubated in the dark at 37 °C for 15 min. Subsequently, 100 µL of stop solution was added to each well to terminate the reaction. The optical density was measured at 450 nm using a microplate reader, with blank wells serving as the baseline for zero adjustment.

### Flow cytometry (FCM)

Following completion of M2 macrophage polarisation, cells were harvested by centrifugation at 1000 rpm for 5 min and washed with PBS. The supernatant was removed, and the cell pellet was resuspended in 90 µL of PBS to achieve a final concentration of 1 × 10⁵ cells/mL. CD11b and CD206 antibodies were added to the suspension and mixed thoroughly. Samples were incubated at 37 °C for 30 min in the dark. After incubation, 400 µL of PBS was added to each tube. Macrophage phenotypes were then analyzed utilizing FCM.

### Immunofluorescence (IF) staining and semi-quantitative analysis

Cell samples were air-dried and fixed overnight in 4% paraformaldehyde. After fixation, samples were rinsed with PBS and incubated with 3% hydrogen peroxide-methanol solution for 10 min at room temperature to block endogenous peroxidase activity. After additional PBS washes, sections were incubated with 50% BSA for 20 min at room temperature to reduce nonspecific binding. Primary antibodies targeting CCL17 (bs-2453R, Bioss), CCL18 (DF9914, Affinity), CD206 (18704-1-AP, Proteintech), and CD11b (66519-1-Ig, Proteintech) were added dropwise (50 µL per section), and samples were incubated for 2 h in a humidified chamber at 37 °C. Following incubation, samples were washed with PBS and incubated with 50 µL of FITC-conjugated secondary antibody for 1 h at 37 °C in the dark. DAPI staining solution (50 µL) was added to each section and incubated for 5 min at room temperature in the dark. Fluorescence signals were observed under a fluorescence microscope. Three random fields per section were selected for semi-quantitative analysis of fluorescence intensity.

### Western blot (WB)

Protein expression was assessed by WB. Cell sample proteins were extracted, and protein concentrations were determined, followed by electrophoresis and membrane transfer. Membranes were incubated overnight at 4 °C with primary antibodies specific to IL-10 (60269-1-Ig, Proteintech), JAK2 (AF6022, Affinity), STAT3 (10253-2-AP, Proteintech), VEGF (66828-1-Ig, Proteintech), VEGFR (26415-1-AP, Proteintech), and CD34 (14486-1-AP, Proteintech). After washing, membranes were incubated with appropriate secondary antibodies for 2 h at room temperature. Protein bands were visualized utilizing the ECL Chemiluminescence Kit (KGC4902-20, KeyGEN Biotech), imaged with the ChemiDoc MP Imaging System, and analyzed by grayscale densitometry using Gel-Pro32 software.

### Real time-quantitative polymerase chain reaction (RT-qPCR)

Total RNA was extracted utilizing TRIzol reagent, and cDNA was synthesized for reverse transcription PCR using a cDNA strand synthesis kit (RR036B, TaKaRa). RT-qPCR was performed utilizing the TB Green^®^*Premix Ex Taq*™ II (Tli RNaseH Plus) kit (RR820A, TaKaRa). Primer sequences for RT-qPCR are shown in Table 2.

**Table 2:** PCR primer sequences

Gene	Species	Primers	Sequences
JAK 2	Human	Forward	5^,^ -TCTCAGATATGCAAGGGTATGG-3^,^
		Reverse	5^,^ -TGGGACTTTCACCAGGTTCTT-3^,^
STAT 3	Human	Forward	5^,^ -GAAGAGGCGGCAACAGATT-3^,^
		Reverse	5^,^ -TGGGGTCCCCTTTGTAGGA-3^,^
COL2	Human	Forward	5^,^ -AATAGCAGGTTCACGTACACTG-3^,^
		Reverse	5^,^ -GTACTCGATAACAGTCTTGCCC-3^,^
Aggrecan	Human	Forward	5^,^ -AATGAGCCCTATGGAGATGACA-3^,^
		Reverse	5^,^ -GGTAGTCTCCATCCATGTCCAT-3^,^
MMP-3	Human	Forward	5^,^ -TCCGACACTCTGGAGGTGATGC-3^,^
		Reverse	5^,^ -TCGGGATGCCAGGAAAGGTTCT-3^,^
MMP-7	Human	Forward	5^,^ -GAACAGGCTCAGGACTATCTCA-3^,^
		Reverse	5^,^ -CATCTCCTTGAGTTTGGCTTCT-3^,^
GAPDH	Human	Forward	5^,^ -CAAATTCCATGGCACCGTCA-3^,^
		Reverse	5^,^ -AGCATCGCCCCACTTGATTT-3^,^

### Statistical analysis

SPSS 26.0 was used for data analysis. Data with normal distribution were expressed as mean ± standard deviation($\bar {x} \pm s$). Two-group comparisons were conducted using independent-sample t-tests. One-way analysis of variance (ANOVA) was used for comparisons among multiple groups. *P* < 0.05 was used to indicate that the difference was significant.

## Results

### Macrophage infiltration and microvascular invasion in human degenerated NP

**Fig. 1:**
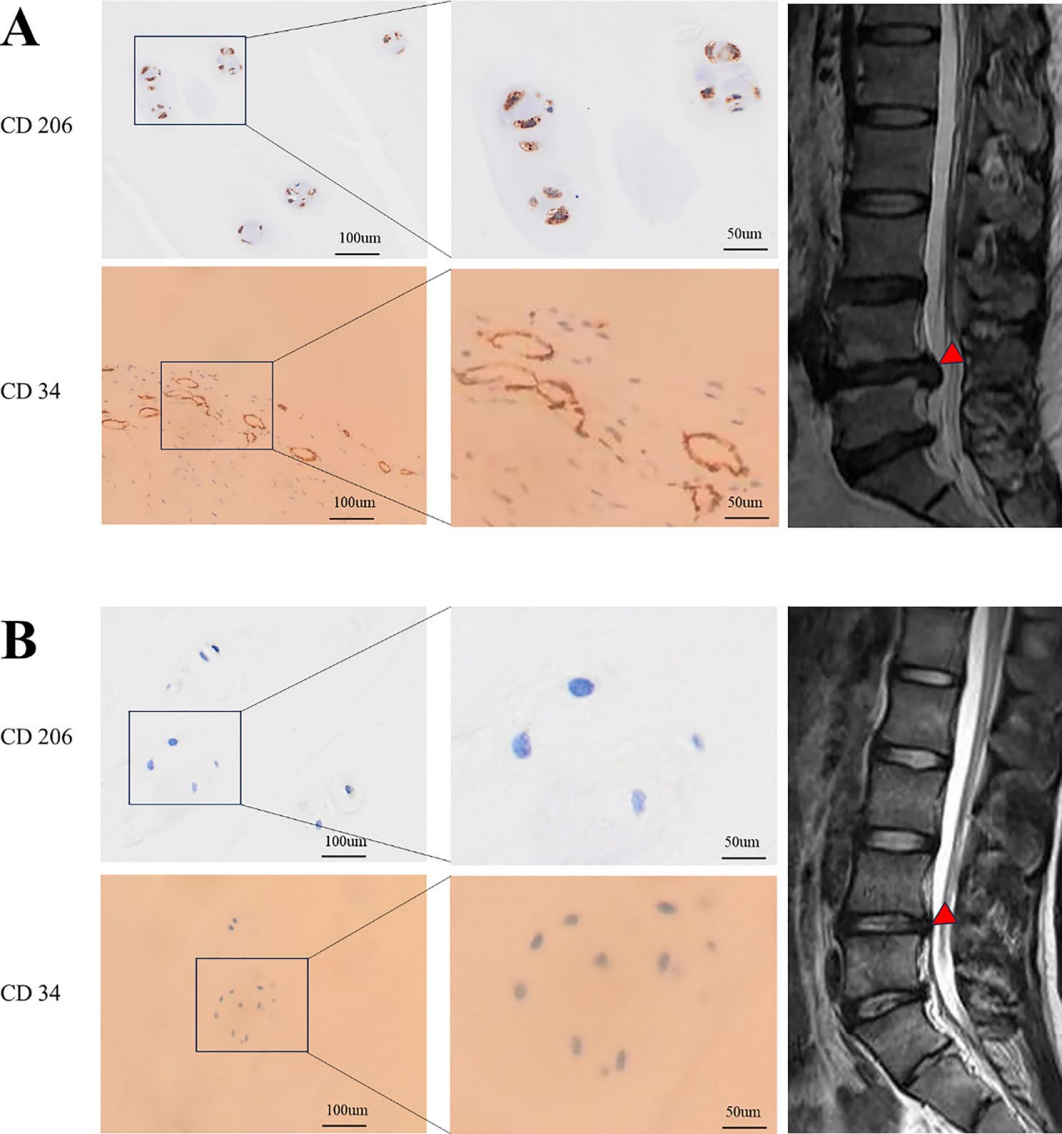
Differences between macrophage infiltration and microvascular invasion in degenerated NPs

In the severely degenerated NP tissues characterized by the “black disc” sign, a marked reduction in NP cell number was observed, along with pronounced degradation and disorganization of collagen type II (COL2) and aggrecan. IHC staining identified clusters of CD206-positive macrophages and CD34-positive structures indicative of pathological microvascular lumen formation and endothelial cell infiltration at the NP margins (Fig. 1A). In contrast, mildly degenerated NP tissues lacking the “black disc” sign exhibited dispersed NP cells with relatively preserved COL2 and aggrecan. These samples did not detect CD206- and CD34-positive cells (Fig. 1B).

**A**: NP tissue from a 46-year-old male patient with modified Pfirrmann grade 6 degeneration. IHC staining revealed clusters of CD206-positive macrophages and CD34-positive endothelial cells, with evident formation of pathological microvascular lumens. **B**: NP tissue from a 17-year-old male patient with modified Pfirrmann grade 2 degeneration. No CD206-positive macrophages or CD34-positive endothelial cells were detected.

### Screening of optimal concentrations for LPS-induced degeneration of NP cells

**Fig. 2:**
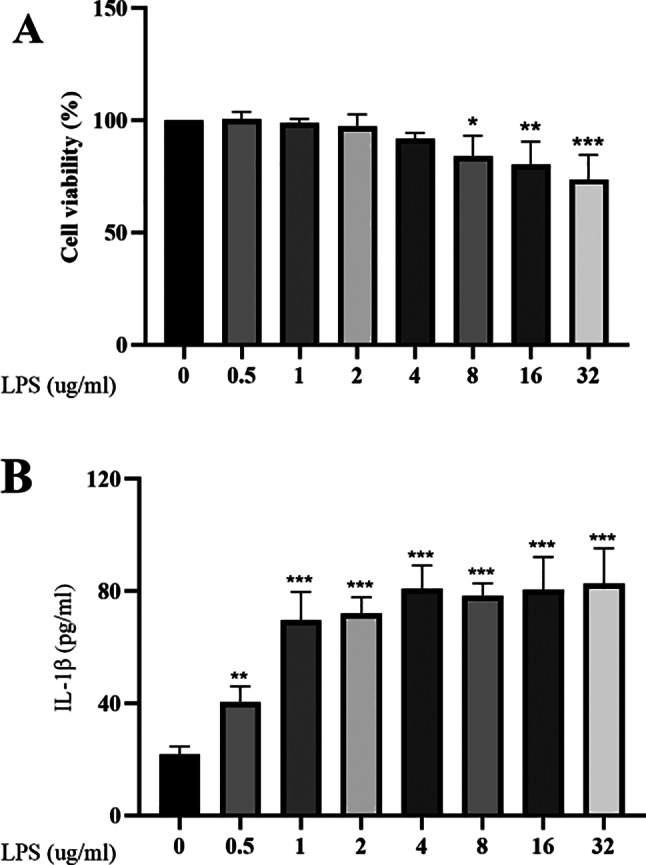
Screening for optimal intervention concentration of LPS. **A**: CCK8 assay for cell viability; **B**: ELISA for changes in IL-1β concentration. *P<0.05; **P<0.01; ***P<0.001

NP cells were treated with varying concentrations of LPS to determine the optimal dose for inducing degeneration. Cell viability was assessed using the CCK-8 assay, and interleukin-1β (IL-1β) expression, a marker of degeneration, was quantified by ELISA. A significant decrease in cell viability was observed when LPS concentration reached 8 µg/mL (Fig. 2A). IL-1β expression increased markedly at 0.5 µg/mL, indicating the onset of degeneration. Expression levels continued to rise with increasing LPS concentrations and peaked at 4 µg/mL, followed by a gradual decline and stabilization at higher concentrations (Fig. 2B). To induce degeneration while minimizing cytotoxicity, 4 µg/mL was selected as the optimal LPS concentration for subsequent experiments.

### Polarisation of M2 macrophages

**Fig. 3:**
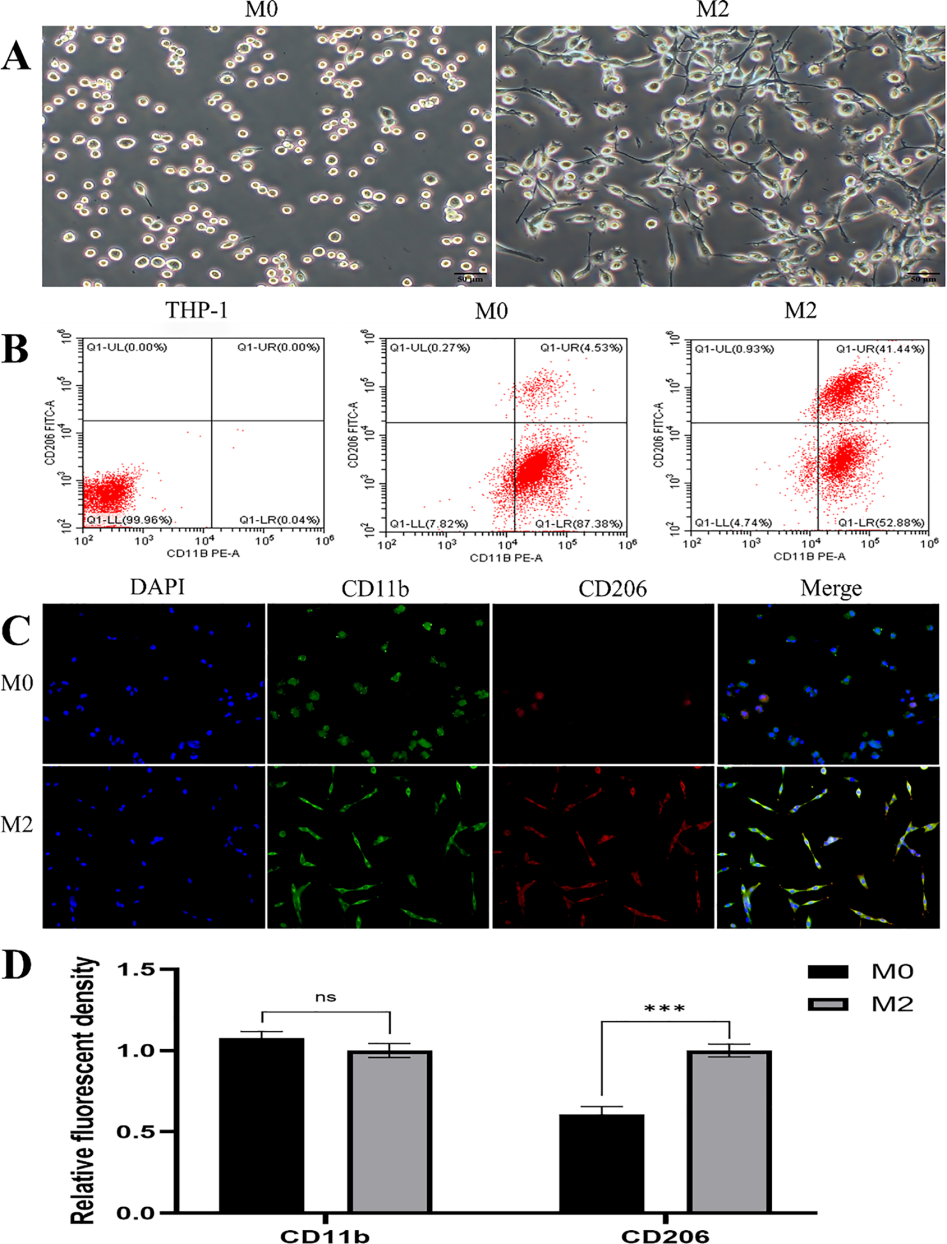
Macrophage polarisation phenotype detection. **A**: Morphological characteristics of M0 and M2 macrophages under light microscopy; **B**: FCM analysis of CD11b and CD206 expression; **C**: IF staining of CD11b and CD206 in M0 and M2 macrophages; **D**: Semi-quantitative analysis of CD11b and CD206 detected by IF staining

THP-1 cells were used as the precursor cell line for macrophage differentiation. Morphological analysis showed that M0 macrophages exhibited a round or oval shape, small nuclei, uniform appearance, and limited pseudopodia. Following IL-4 and IL-13 stimulation, M2 macrophages displayed an enlarged, elongated morphology with uniform cell bodies and extended pseudopodia lacking bifurcation (Fig. 3A). FCM demonstrated a significant increase in CD11b expression after PMA treatment, confirming successful differentiation of THP-1 cells into M0 macrophages. Subsequent stimulation with IL-4 and IL-13 led to a marked upregulation of CD206, co-expressed with CD11b, indicating successful polarisation into the M2 phenotype (Fig. 3B). IF staining further supported these findings, showing enhanced co-expression of CD11b and CD206 in M2 macrophages compared to M0 cells (Fig. 3C and D).

### Expression of macrophage markers in degenerated NP

**Fig. 4:**
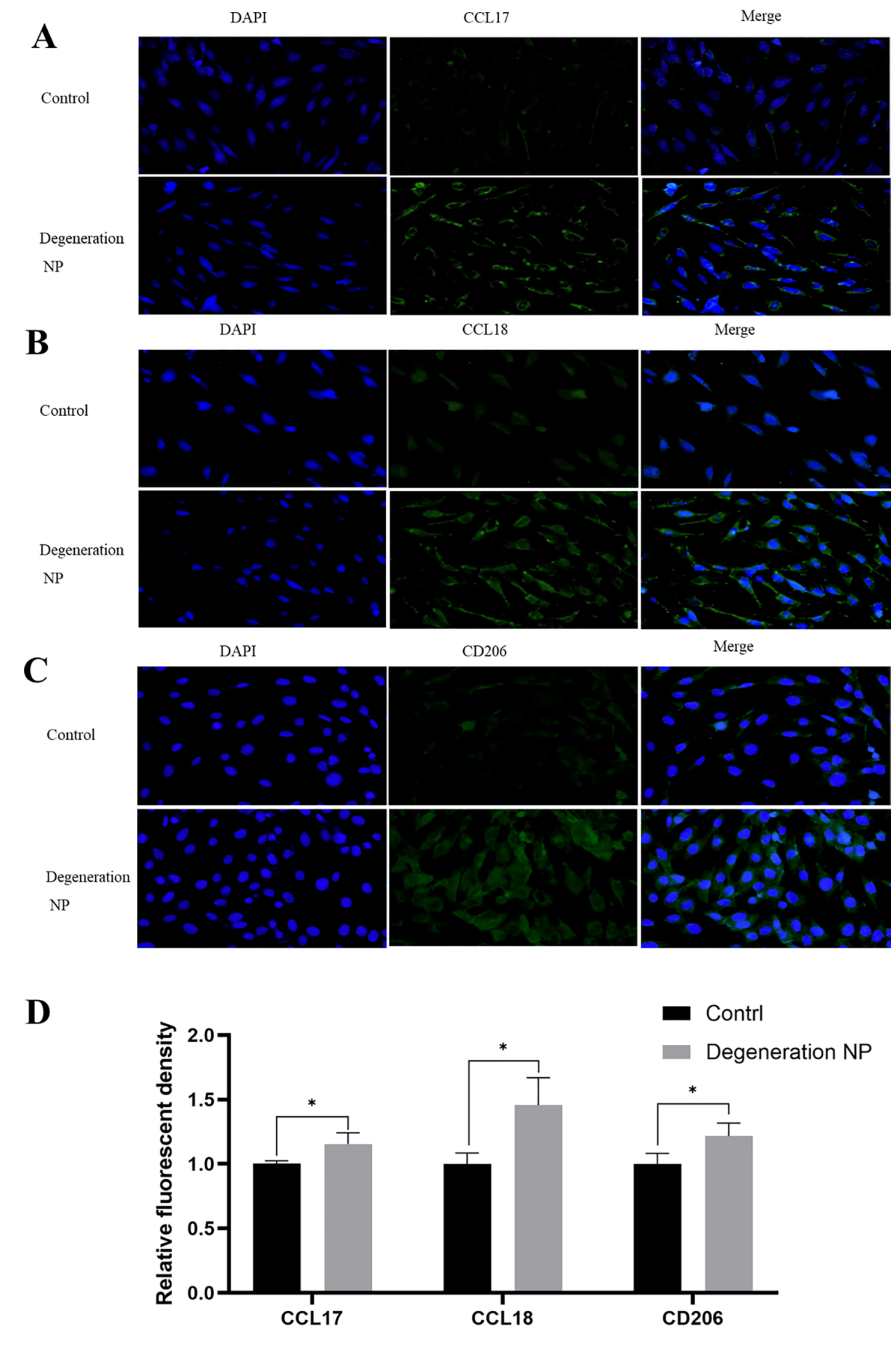
Expression of M2 macrophage markers (CCL 17, CCL18, CD206) detected by IF staining in degenerated NP. **A**: CCL17 expression detected by IF staining; **B**: CCL18 expression detected by IF staining; **C**: CD206 expression detected by IF staining; **D**: Semi-quantitative analysis of fluorescence intensity for CCL17, CCL18, and CD206. *P<0.05

NP cells were treated with 4 µg/mL lipopolysaccharide (LPS) to induce degeneration, and the expression of M2 macrophage markers (CCL17, CCL18, CD206) was detected by IF staining and semi-quantitative analysis. The results suggested that the expression of CCL17, CCL18 and CD206 in degenerated NP cells was higher than in the control group (Fig. 4A-D).

### M2 macrophages promote pathological microangiogenesis and ECM metabolic imbalance in the NP by secreting IL-10

**Fig. 5:**
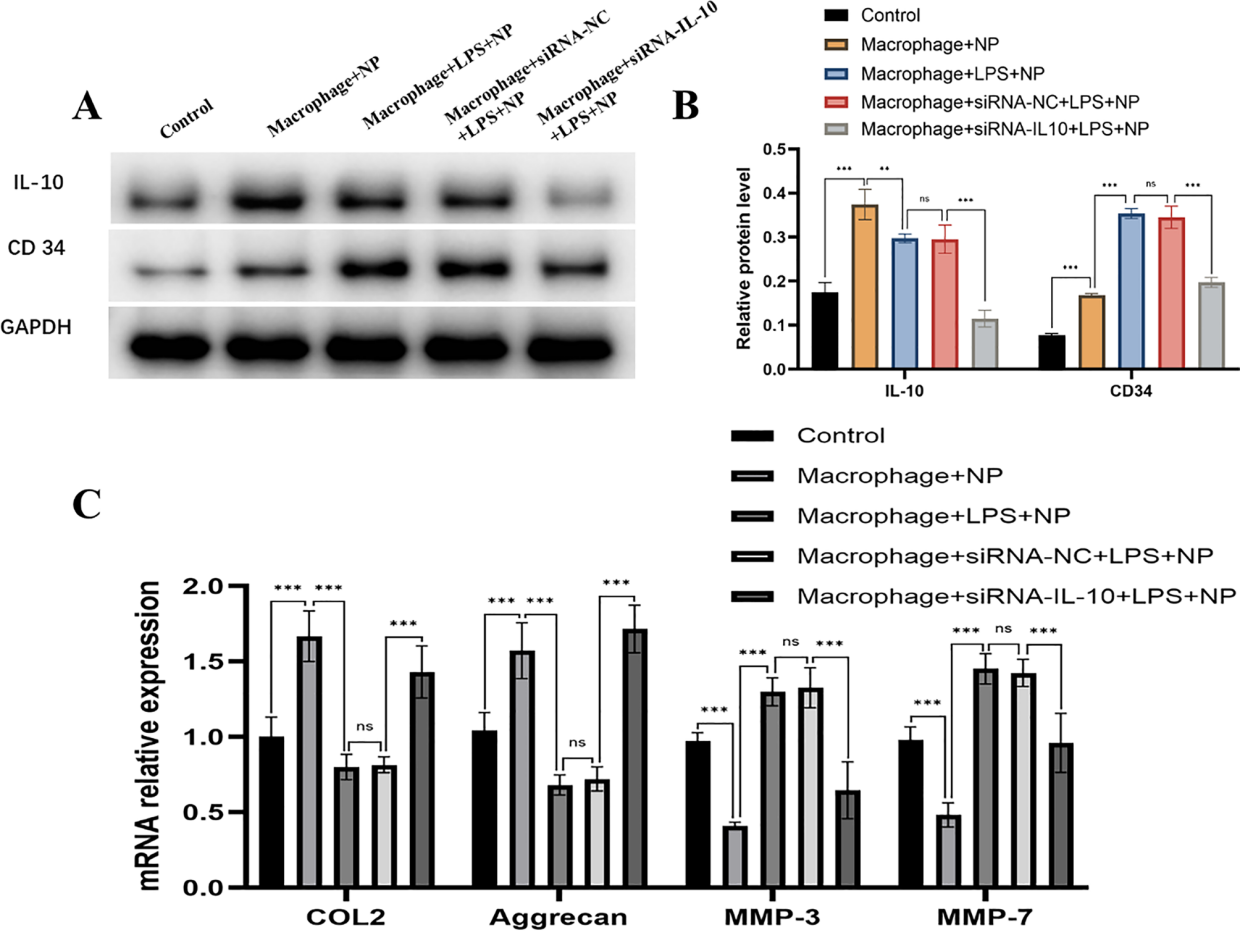
Effect of IL-10 secreted by M2 macrophages co-cultured with NP cells on pathological microangiogenesis and ECM metabolism. **A**: WB detection of the difference in protein expression of IL-10 and CD34 in NP cells; **B**: quantitative analysis of protein expression of IL-10 and CD34; **C**: RT-qPCR detection of COL2, aggrecan, MMP-3, and MMP-7 mRNA expression in each group.nsP>0.05;**P<0.01; ***P<0.001

WB analysis revealed that co-culture of M2 macrophages with normal NP cells led to a significant increase in IL-10 expression compared to the normal NP control. When co-cultured with degenerated NP cells, IL-10 expression was significantly lower than in the normal co-culture group. Further reduction in IL-10 levels was observed following siRNA-mediated knockdown, indicating effective silencing of IL-10. CD34 expression was higher in M2 macrophages co-cultured with NP cells compared to the blank control group. CD34 expression further increased when M2 macrophages were co-cultured with degenerated NP cells, but was reduced after IL-10 knockdown (Fig. 5A and B). These findings suggest that IL-10 promotes pathological microangiogenesis in the NP microenvironment.

To examine the role of IL-10 in ECM metabolism, RT-qPCR was used to quantify the expression of key anabolic genes (COL2 and aggrecan) and catabolic genes (MMP-3 and MMP-7). Co-culture of M2 macrophages with normal NP cells led to increased expression of COL2 and aggrecan compared to the blank control. In contrast, co-culture with degenerated NP cells resulted in marked downregulation of COL2 and aggrecan. Silencing of IL-10 significantly restored the expression of COL2 and aggrecan compared to the siRNA negative control and the blank control group. Conversely, expression of MMP-3 and MMP-7 was reduced following co-culture with normal NP cells but was significantly elevated in the degenerated co-culture group. siRNA-mediated silencing of IL-10 resulted in a marked decrease in MMP-3 and MMP-7 expression in degenerated NP cells (Fig. 5C**)**. These results indicate that IL-10 secreted by M2 macrophages contributes to ECM degradation by downregulating anabolic genes s and upregulating catabolic genes in the degenerated NP.

### The JAK 2/STAT 3 pathway is an important downstream signaling pathway for M2 macrophages to secrete IL-10 and exert a biological effect in nucleus pulposus cells

**Fig. 6:**
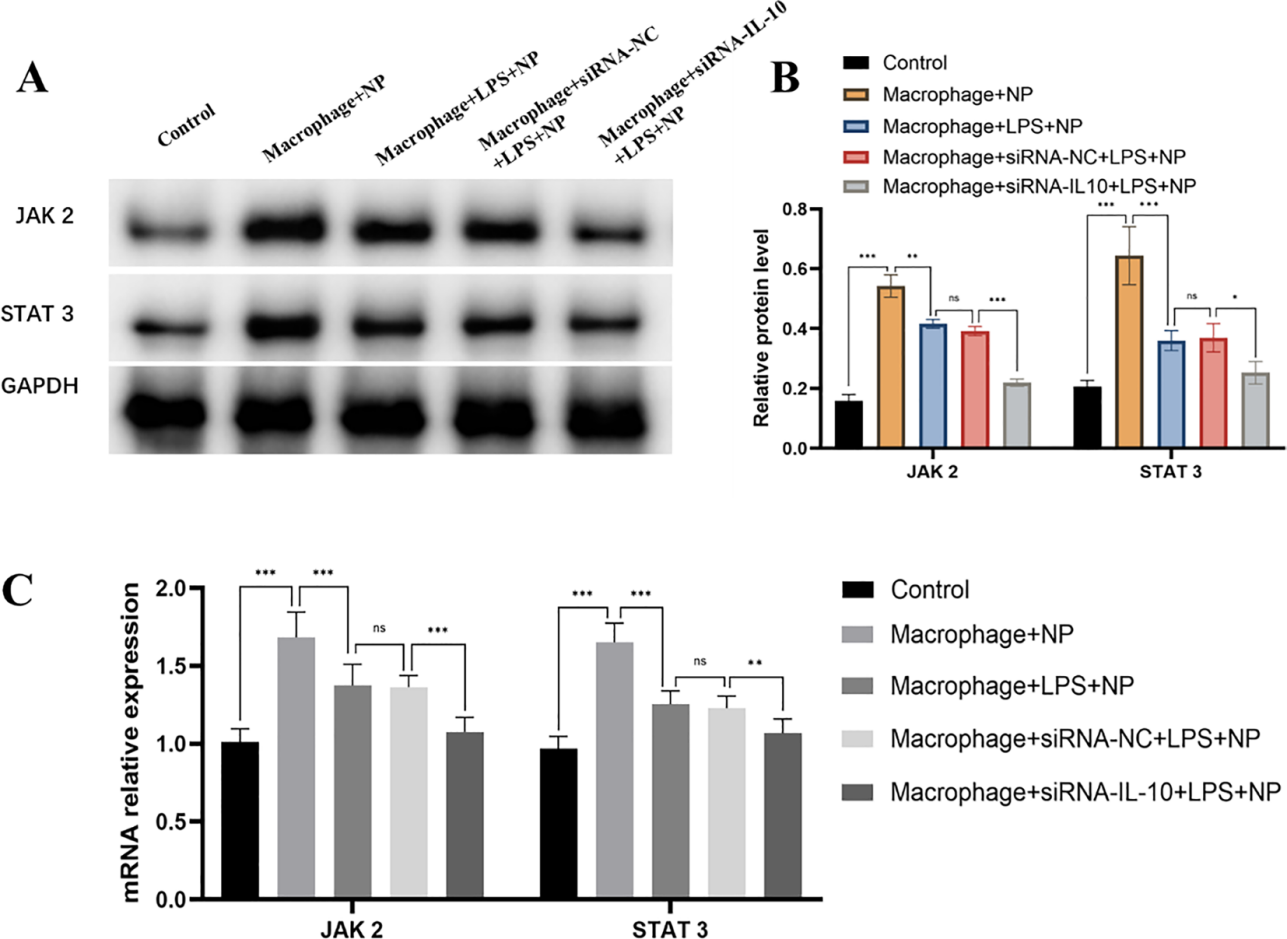
Effect of silencing IL-10 on the JAK 2/STAT 3 pathway after co-culture of M2 macrophages with NP cells. **A**: WB analysis of JAK2 and STAT3 protein expression in NP cells; **B**: Quantitative analysis of JAK2 and STAT3 protein levels; **C**: RT-qPCR analysis of JAK2 and STAT3 mRNA expression. ^ns^P>0.05; *P<0.05; **P<0.01; ***P<0.001

WB and RT-qPCR were performed to evaluate JAK2 and STAT3 expression in NP cells under different co-culture conditions. Co-culture with M2 macrophages significantly upregulated the protein expression of JAK2 and STAT3 compared to the blank control group, indicating that M2 macrophages activate this pathway in NP cells. In contrast, co-culture with degenerated NP cells resulted in lower expression levels of both proteins relative to the normal co-culture group. Silencing of IL-10 using siRNA led to a marked decrease in JAK2 and STAT3 expression compared to both the siRNA negative control and the degenerated co-culture group (Fig. 6A and B). Similar results were found by RT-qPCR (Fig. 6C). These findings suggest that IL-10 secreted by M2 macrophages may exert its regulatory effects on NP cells primarily through activation of the JAK2/STAT3 pathway.

### JAK2/STAT3 pathway upregulates VEGF/VEGFR expression in degenerated NP cells, induces pathological microangiogenesis and thus promotes ECM degradation

**Fig. 7:**
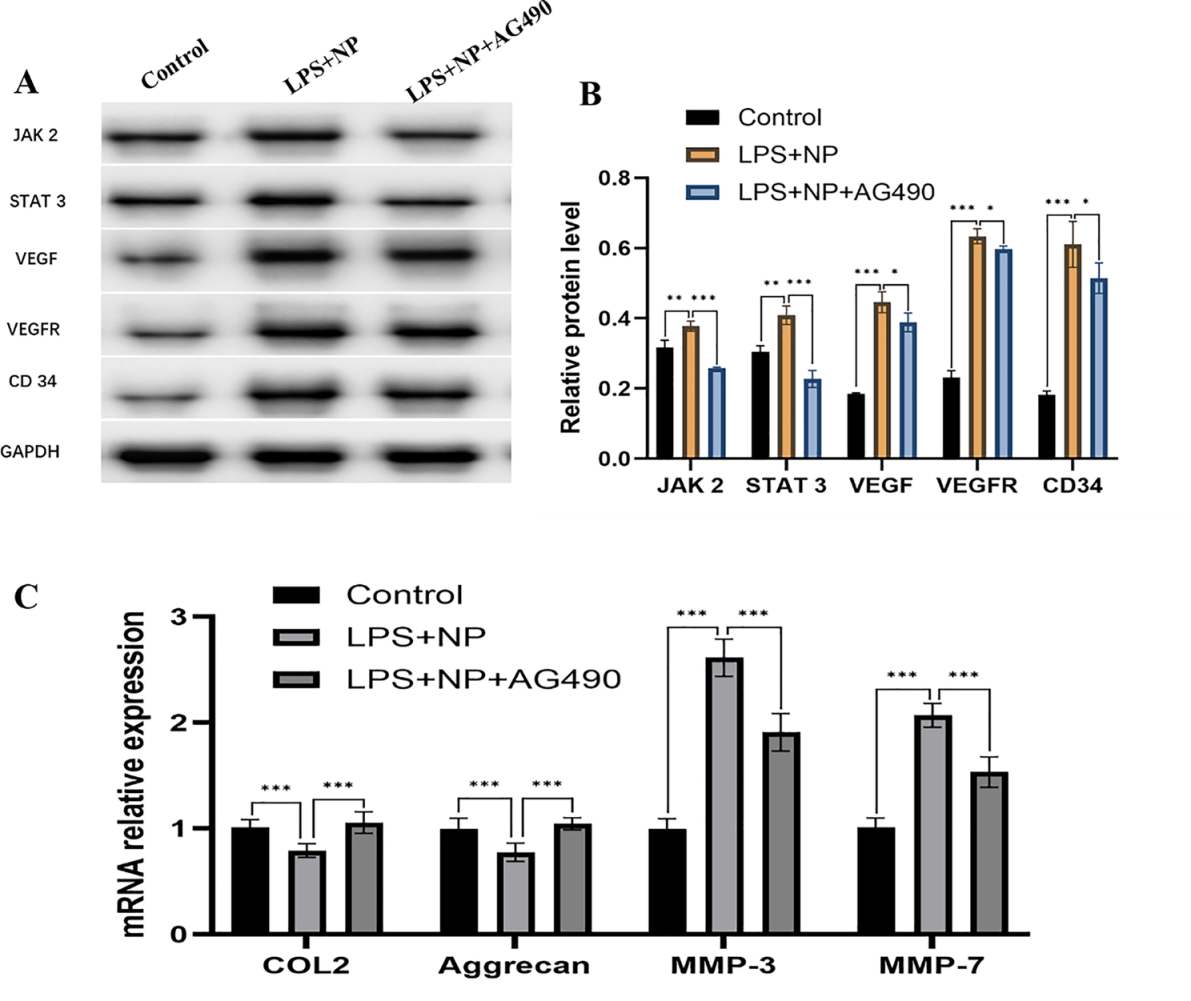
Effect of the JAK 2/STAT 3 pathway on pathological microangiogenesis and ECM metabolism in degenerated NP. **A**: WB analysis of JAK2, STAT3, VEGF, VEGFR, and CD34 protein expression in normal NP cells, degenerated NP cells, and degenerated NP cells treated with the JAK2/STAT3 inhibitor AG490; **B**: Quantitative analysis of protein expression levels for JAK2, STAT3, VEGF, VEGFR, and **C**D34; C: RT-qPCR analysis of mRNA expression levels of COL2, aggrecan, MMP-3, and MMP-7. *P<0.05; **P<0.01; ***P<0.001

In degenerated NP cells, protein expression of JAK2 and STAT3 was significantly elevated compared to the blank control group. Inhibition of the JAK2/STAT3 pathway using AG490 resulted in a marked decrease in both proteins, indicating that JAK2 and STAT3 are upregulated and functionally active in degenerative conditions. Consistent with these findings, the expression of VEGF, VEGFR, and CD34, key markers of angiogenesis, was increased in degenerated NP cells and significantly suppressed following JAK2/STAT3 pathway inhibition (Fig. 7A and B). These results suggest that activation of JAK2/STAT3 signaling drives the upregulation of VEGF/VEGFR and contributes to pathological microangiogenesis in the degenerative NP microenvironment.

RT-qPCR analysis further demonstrated that the mRNA levels of COL2 and aggrecan were significantly downregulated in degenerated NP cells, while inhibition of JAK2/STAT3 by AG490 restored their expression. Conversely, MMP-3 and MMP-7 mRNA expression was significantly elevated under degenerative conditions and significantly reduced following pathway inhibition (Fig. 7C). These findings confirm that activation of JAK2/STAT3 contributes to ECM degradation by downregulating anabolic genes and upregulating catabolic genes in degenerated NP cells.

## Discussion

The present study identified the infiltration of M2 macrophages in degenerated NP tissues, accompanied by pathological microangiogenesis. Co-culture experiments demonstrated that IL-10 secretion from M2 macrophages promotes abnormal neovascularization within the NP, contributing to an imbalance in ECM metabolism. In degenerated NP cells, IL-10 induced pathological microangiogenesis by upregulating VEGF and VEGFR expression through activation of the JAK2/STAT3 signaling pathway, ultimately accelerating ECM degradation and exacerbating IDD.

IDV induced by pathological angiogenesis has been recognized as a key factor in compromising the immune-privileged status of the NP. This process intensifies the inflammatory milieu within the IVD, promotes apoptosis of NP cells, and contributes to the progression of IDD [14]. Macrophages have a crucial role in IDD, and when the NP shows the ‘black disc’ sign, it represents a breakdown of its immune barrier and establishes immune crosstalk with macrophages [15]. After the successful establishment of crosstalk, macrophages may promote microvascular invasion through several mechanisms, including the induction of NP cell apoptosis, direct secretion of VEGF, and ECM degradation via MMPs, thereby accelerating IDV [16–18]. We also detected macrophages and pathological microvessels in degenerated NP tissue, indicating that a potential role for macrophages in promoting aberrant neovascularization and accelerating the progression of IDD. Although earlier studies characterized M2 macrophages as anti-inflammatory and reparative cells capable of mitigating disc degeneration [19], more recent findings indicate a strong association between M2 macrophage infiltration and the severity of NP degeneration. M2 macrophages may contribute to degeneration through multiple mechanisms, including disruption of ECM homeostasis, ultimately leading to a maladaptive “pathological repair” process. Such a failed healing response compromises the structural and functional integrity of the NP and further exacerbates IDD [15, 20–22].

The origin of macrophages within the NP remains a subject of ongoing debate. Some studies have proposed that [15, 23] NP-infiltrating macrophages primarily originate from the circulating monocyte-phagocyte system, commonly referred to as bone marrow-derived macrophages. Other investigations [24] have suggested that circulating monocytes may enter the IVD during embryonic development and differentiate into tissue-resident macrophages, which remain quiescent until activated during IDD. In the present study, significantly increased fluorescence expression of CD206, CCL17, and CCL18 was observed in degenerated NP cells compared to normal controls, indicating that degenerated NP cells may temporarily exhibit an “M2-like” phenotype. Such behavior suggests a potential capacity to secrete M2-associated cytokines and contribute to the progression of IDD. However, degeneration is not sustained solely by the secretory activity of NP cells. Upon the breakdown of the immune barrier and establishment of immune crosstalk, bone marrow-derived macrophages and associated inflammatory mediators infiltrate the NP. These immune components drive the inflammatory cascade and pathological repair responses that exacerbate IDD through the unresolved conflict between inflammation and pathological regeneration [25].

Induction of endothelial cell migration and promotion of microangiogenesis are key functions of M2 macrophages, which contribute to wound healing and tissue repair processes [26–28]. In the present study, IL-10 secreted by M2 macrophages exhibited a pronounced pro-angiogenic effect in degenerated NP cells. IL-10, a cytokine typically associated with the M2 macrophage phenotype [29], has been reported to exert divergent effects on angiogenesis. While earlier studies suggested an inhibitory role, recent evidence has demonstrated a strong pro-angiogenic capacity under specific physiological and pathological conditions [30, 31]. Niu et al. [32] reported that IL-10 is critical in regulating angiogenesis during tissue and organ development by enhancing local blood perfusion, primarily by activating STAT3 signaling. Liu et al. [33] further demonstrated that M2 macrophages promote angiogenesis during tissue repair, with IL-10 stimulating neovascularization by reinforce M2 macrophage polarization, thereby improving local blood circulation for ischaemic muscle repair.

Upon binding to its receptor, IL-10 mediates receptor dimerization, activating and recruiting JAKs. Activated JAKs induce phosphorylation and dimerization of STATs, which subsequently translocate to the nucleus and regulate gene transcription [34]. The JAK2/STAT3 signaling pathway is critical in angiogenesis-related disorders, with STAT3-dependent VEGF expression reported in human tumor cells [35]. Wang et al. [36] further demonstrated that JAK2 activity regulates STAT3-mediated VEGF expression, significantly enhancing vascular permeability. The STAT3/VEGF axis has been shown to exert strong pro-angiogenic effects in various disease contexts, contributing to disorganized neovascularization and local fibrotic remodeling [37]. Activation of this axis also promotes the proliferation and migration of vascular endothelial progenitor cells, further supporting localized microangiogenesis [38]. VEGF expression in IDD has been closely linked to disease severity, with elevated VEGF levels marking the onset of degeneration. Although previous studies have noted concurrent upregulation of IL-10, JAK2, and STAT3 in the presence of M2 macrophages, no prior work has clarified the mechanistic relationship between IL-10 secretion by M2 macrophages and JAK2/STAT3 activation in degenerated NP cells [39]. In this study, activation of the JAK2/STAT3 pathway was found to be primarily regulated by IL-10 secreted by M2 macrophages. This signaling cascade enhances VEGF–VEGFR interaction in degenerated NP cells, inducing vascular endothelial cell migration and activation, thereby promoting pathological microangiogenesis. The resulting IDV contributes to aberrant tissue repair within the IVD and accelerates the progression of IDD.

Consistent with the findings of Li et al. [40], We found that M2 macrophages did not inhibit COL2 and aggrecan expression in normal NP cells. In contrast, M2 macrophages upregulated MMPs in degenerated NP cells, promoting ECM degradation and accelerating IDD progression. However, differently, we found that pathological microangiogenesis in the NP induced by M2 macrophages is another key factor promoting ECM degradation. Activation of the JAK2/STAT3 pathway by IL-10 secreted from M2 macrophages enhanced endothelial cell activation and migration through the STAT3/VEGF axis, leading to pathological neovascularization within the NP. The formation of these aberrant vessels provided an entry route for MMP infiltration into NP tissue. Concurrently, neovascularization established a connection between the NP and systemic circulation, disrupting the local microenvironment and formating an oxygen-rich state that impaired COL2 and aggrecan synthesis, ultimately disturbing ECM homeostasis [41]. Suzuki et al. [42] reported that the JAK2/STAT3 pathway is a major regulator of MMP expression. As a member of the MMP family, MMP-3 actively degrades various ECM components [43]. Sun et al. [44] identified a potential causal relationship between elevated plasma MMP-3 levels and the risk of IDD, suggesting a critical role for MMP-3 in IDD progression. MMP-7 also contributes to ECM degradation and has been shown to accumulate in regions of IVD degeneration, where it facilitates matrix breakdown and accelerates ECM deterioration [45]. Therefor, the secretion of IL-10 by M2 macrophages promotes pathological microangiogenesis and facilitates the infiltration of MMP-3 and MMP-7 into the NP. Upregulation of these matrix-degrading enzymes plays a central role in ECM degradation and the disruption of metabolic homeostasis in the NP. In addition to mediating ECM breakdown, MMP-3 and MMP-7 contribute to microvascular invasion by remodeling the matrix. MMP-7, functions as an angiogenic inducer, enhancing endothelial cell proliferation and promoting neovessel formation, which enables deeper vascular infiltration into NP tissue [46, 47]. In IDD, microvascular invasion driven by activation of the JAK2/STAT3 pathway accelerates the degradation of COL2 and aggrecan and induces the expression of MMP-3 and MMP-7. These molecular events contribute to metabolic dysregulation of the ECM, structural disintegration of the NP, and progressive loss of IVD function, thereby exacerbating the development of IDD.

## Conclusion

Infiltration of M2 macrophages and pathological microvascular proliferation are prominent features in degenerated NP tissue. Degenerated NP cells also exhibit the capacity to express macrophage-associated markers. Within this degenerative microenvironment, M2 macrophages secrete IL-10, activating the JAK2/STAT3 signaling pathway, leading to upregulating VEGF and VEGFR expression and promoting pathological angiogenesis in the NP. This cascade downregulates anabolic genes (COL2 and aggrecan), while simultaneously upregulating catabolic genes (MMP-3 and MMP-7). The resulting disruption of ECM metabolic homeostasis contributes to the structural deterioration of the IVD and accelerates the progression of IDD.

## Data Availability

The dataset(s) supporting the conclusions of this article are included within the article.

## Abbreviations

IVD: Intervertebral disc
IDD: Intervertebral disc degeneration
IDV: Intervertebral disc vascularisation
NP: Nucleus pulposus
IL: Interleukin
JAK: Janus kinase
STAT: Signal transducer and activator of transcription
IHC: Immunohistochemistry
CCK-8: Cell Counting Kit-8
ELISA: Enzyme-linked immunoSorbent assay
FCM: Flow cytometry.
IF: Immunofluorescence
WB: Western blot
RT-qPCR: Real time-quantitative polymerase chain reaction
ECM: Extracellular matrix
VEGF: Vascular endothelial growth factor
VEGFR: Vascular endothelial growth factor receptor
COL2: Type II collagen
MMP: Matrix metalloproteinase
VEGF: Vascular endothelial growth factor
